# Prevalence and associated factors of self-medication among pregnant women in Sodo Town, Southern Ethiopia

**DOI:** 10.3389/fmed.2024.1379706

**Published:** 2024-10-09

**Authors:** Mesfin Abebe, Silenat Gashaw, Dinkalem Getahun, Wudit Wassu, Tiruye Menshaw, Serawit Lakew

**Affiliations:** ^1^Department of Midwifery, College of Medicine and Health Science, Dilla University, Dilla, Ethiopia; ^2^Department of Midwifery, College of Medicine and Health Science, Wolaita Sodo University, Wolaita Sodo, Ethiopia; ^3^School of Nursing, College of Medicine and Health Sciences, Arba Minch University, Arba Minch, Ethiopia; ^4^Department of Midwifery, College of Medicine and Health Science, Wachamo University, Hosanna, Ethiopia; ^5^Department of Pediatrics and Child Health Nursing, College of Medicine and Health Science, Debre Markos University, Debre Markos, Ethiopia

**Keywords:** self-medication practice, magnitude, associated factors, pregnant women, Ethiopia

## Abstract

**Background:**

Self-medication is a widespread practice among pregnant women worldwide, with 44.55% practicing it. However, it may lead to incorrect diagnosis, inappropriate treatment, and failure to recognize contraindications. The adverse effects of self-medication are often unknown, and they pose potential risks to maternal and fetal health. This study aimed to address the gap in research on the prevalence and associated factors of self-medication among pregnant women in Southern Ethiopia, and to promote effective strategies and interventions.

**Methods:**

A community-based cross-sectional study was conducted among 425 pregnant women in Sodo Town from April to June 2021. Participants were enrolled using a two-stage sampling technique. Data were collected using a structured questionnaire and face-to-face interviews. The association between the outcome and independent variables was assessed through bivariate logistic regression analysis. Additionally, multivariable logistic regression analysis was carried out, including variables with an adjusted odds ratio (AOR) accompanied by a 95% confidence interval (CI) and a *p*-value of less than 0.05, which was considered statistically significant.

**Results:**

In this study, pregnant women aged 18–39 years participated with a 100% response rate. The study found that the prevalence of self-medication during pregnancy was 20.5%, with 8.2% using herbal medicine and 12.3% using conventional medicine. A history of self-medication and first-and second-trimester pregnancy were significantly associated with self-medication. A history of self-medication (AOR = 6.31, 95% CI: 4.89, 9.91) and being in the first trimester of pregnancy (AOR = 3.47, 95% CI: 1.63, 7.38) or second trimester of pregnancy (AOR = 2.14, 95% CI: 1.12, 4.08) were associated with a higher likelihood of self-medication.

**Conclusion:**

The study found that 20.5% of pregnant women engaged in self-medication. It also identified that a previous history of self-medication and gestational age were factors associated with self-medication. These findings underscore the need for targeted educational programs and policy interventions to mitigate the risks associated with self-medication during pregnancy, particularly in the early stages of gestation.

## Background

Self-medication refers to using medication without a prescription from a healthcare provider for self-identified health issues (1). Self-medication can lead to incorrect self-diagnosis, which, in turn, can result in inappropriate choice of treatment. This practice also increases the risk of failing to recognize specific contraindications, warnings, and precautions (2). Self-medication is widely practiced globally, including in both developed and developing countries. It is reported that 44.55% of pregnant women worldwide practice self-medication (3). In Africa, self-medication prevalence varies widely, with reported rates ranging from 16% in South Africa to as high as 85% in certain parts of Nigeria (4). A study in France showed that 41.50% of pregnant women practiced self-medication (5). In Iran, a systematic review and meta-analysis study revealed a self-medication prevalence of 32% (6). In Sweden, a study in 2017 found that exposure to antidepressants in the first trimester was associated with a 6.98% increase in preterm birth among offspring compared to non-exposure (7). A systematic review in Ethiopia indicated a self-medication prevalence ranging from 12.80 to 77.10% (4). A recent study in the northern part of Ethiopia reported a prevalence of 44.60% among pregnant women attending antenatal care (8).

The adverse effects of self-medication are often unknown, leading to widespread practice and resulting in severe side effects (1, 9–13). Self-medication among pregnant women poses potential risks to both maternal and fetal health (4, 14). During pregnancy in Ethiopia, ginger is the most commonly consumed herbal medicine, followed by damakasse, garlic, tenaadam, and eucalyptus. Conventional medicines include antibiotics, analgesics, and anthelmintics (8, 15). Despite the existence of the 2019 WHO antenatal care recommendations for self-administered interventions for common physiological symptoms, the strength of these recommendations and the certainty of evidence are not clearly defined. Additionally, no specific educational intervention formats are available, including in Ethiopia (16). While medications like non-steroidal anti-inflammatory drugs may pose a low risk to pregnancy, determining the safety of any drug for pregnant women is challenging (17). Pregnant women often turn to herbal medicines alongside conventional treatments. A WHO survey found that 70–80% of people worldwide rely on herbal medicines for primary healthcare. In Africa, including Ethiopia, pregnant women favor herbal remedies due to their affordability and accessibility (11, 18).

Self-medication can present significant challenges for both individuals and the community, particularly for pregnant women (19, 20). Furthermore, there is insufficient evidence to support routine home visits for pregnant women who experience adverse events due to self-medication (21). In developing countries, including Ethiopia, limited access to healthcare facilities, lack of awareness, and poorly regulated medicines drive pregnant women to self-medicate due to affordability and accessibility (18, 22, 23). Many pregnant women are unaware of the various drugs available in the market that are contraindicated during pregnancy. These medications can have potential impacts on both the mother and the unborn child (23).

In Ethiopia, self-medication among pregnant women may be exacerbated by limited access to health facilities, low socioeconomic status, relatively high illiteracy rates, and a lack of awareness regarding the negative consequences of self-medication (2). Data on the prevalence of self-medication among pregnant women, especially in the Southern Ethiopia region, are limited. Therefore, this study aims to fill the gap in the prevalence of self-medication practices and associated factors among pregnant women in Sodo Town, Southern Ethiopia. The findings will help design effective strategies and interventions to address this issue, raise awareness, and emphasize the importance of seeking proper medical advice during pregnancy. Additionally, the study will contribute to the knowledge of self-medication practices among pregnant women in Ethiopia and serve as a resource for policymakers, healthcare providers, and stakeholders.

## Materials and methods

### Study area, design, and timeframe

A community-based cross-sectional study design was conducted in Sodo Town. Sodo Town is located 332 km from Addis Ababa. According to the Central Statistical Agency of Ethiopia (CSA), Sodo Town has a total population of 76,050 people, with 40,140 male residents and 35,910 female residents. The study was carried out in Sodo Town from April to June 2021.

### Population and inclusion criteria

All pregnant women living in Sodo Town were the source population, whereas all pregnant women found in selected kebeles of Sodo Town during the study period were the study population. Pregnant women who lived for more than 6 months in Sodo Town during the data collection period were included. Pregnant women who were critically ill, who were in labor pain, who had lived for less than 6 months in Sodo Town, and who were unable to communicate during the data collection period were excluded.

### Sample size determination

The sample size was determined using the single population proportion formula, assuming a 95% confidence level, a 5% margin of error, a design effect of 2, and an additional 5% non-response rate. For the dependent variable, the prevalence of self-medication practice during pregnancy was taken as 15.5% based on a study conducted in Goba town (24). After using a 5% non-response rate and a design effect of 2, the final sample size was 425.

### Sampling technique and procedure

A two-stage sampling technique was employed in this study. In the first stage, two sub-cities were chosen out of a total of three using a lottery method. The lists of pregnant women residing in these selected sub-cities were then obtained as the sampling frame. In the second stage, participants were selected from each kebeles in proportion to their size using a simple random sampling technique. A sampling frame was created by listing 701 mothers from the HEW registration book (family folder). Within each kebele, participants were selected using simple random sampling. The number of study units assigned to each kebele was proportional to the number of pregnant women. If there were multiple pregnant women in a selected household, one was chosen through a lottery method. If no eligible respondents were available at the time of the survey, the survey was repeated three times.

### Study variables

#### Dependent variables

Self-medication practice.

#### Independent variables

Sociodemographic characteristics (age, marital status, women’s educational status, husbands’ educational status, religion, occupation, and wealth index). Obstetric-related factors (gravidity, parity, gestational age, presence of ANC visit, time of ANC visit initiation, number of living children, previous history of abortion, and pregnancy-related problems). Prior experience and health facility-related factors (availability of herbal and conventional medications, source of information, illness as minor, and prior experience with medication).

### Operational definition and measurements

Self-medication is the use of conventional medicine or herbal medicines by pregnant women to treat self-recognized disorders without a prescription from a healthcare provider (2).

It is classified as ‘Yes’ if a woman has used any pharmaceutical medications during her pregnancy without any medical prescriptions or “No” if she has never used drugs with therapeutic intent with professional advice/prescription (24). Conventional medicine is medicine that one can obtain from medical doctors, nurses, physical therapists, psychologists, and similar healthcare professionals (18). Herbal medicine is the use of medicinal plants for the prevention and treatment of disease (23).

### Data collection tool, procedure, and quality

A structured questionnaire was developed based on a review of the relevant literature (2, 24, 25). The questionnaire was divided into four sections: sociodemographic, obstetric history, knowledge of self-medication, and practice of self-medication. To ensure its reliability and validity, our survey tool underwent a rigorous validation process, including assessments of content validity and face validity. Additionally, it was pre-tested on 5% (22) of pregnant women who were not part of the study sample. Minor modifications were then made to the questionnaire based on the pre-test results.

Data collection was carried out by three experienced individuals with a Bachelor of Science in Midwifery, two HEWs, and one experienced supervisor. In addition, the principal investigator provided a one-day training session for the data collectors and the data quality supervisor. There was one data quality supervisor responsible for overseeing the data collection process. The supervisor ensured data quality by checking for completeness and accuracy alongside the data collectors. Data quality was maintained through training, appropriate supervision, and the use of proper data collection tools. Continuous supervision was provided to ensure data quality, and the data collectors and the supervisor checked all collected data for completeness daily. If any data were found to be incomplete, the supervisor instructed the data collectors to revisit the respondents to obtain the missing information.

### Data management and data analysis

The data were entered into Epidata version 3.1 and analyzed using SPSS version 24. Descriptive statistics, including frequencies, means, and standard deviations, were reported. Each variable was initially analyzed using a binary logistic regression model. Independent variables with a *p*-value of less than 0.25 were then entered into a multivariable logistic regression model for the final analysis. In the multivariable logistic regression analysis, variables with an adjusted odds ratio (AOR) and a 95% confidence interval (CI), along with a *p*-value of less than 0.05, were considered statistically significant. To ensure model adequacy and check for multicollinearity, the variance inflation factor (VIF) and the Hosmer–Lemeshow test were used, respectively. Finally, the results were presented in both tabular and textual formats.

## Results

### Sociodemographic and economic characteristics of the study participants

This study included 425 pregnant women in total, and the response rate was 100%. In total, 212 (49.9%) of the participants women were aged 25–29 years, with a mean age and standard deviation of 26.11 ± 3.74. The majority of them (*N* = 402, 94.6%) were married, and 162 (38.1%) women were housewives. The majority of the pregnant women attended primary school (*N* = 156, 36.7%). The most common religion of the participants was protestant (*N* = 201, 47.3%). The majority of the respondents 112 (26.4%) were grouped under the poor wealth index (Table 1).

**Table 1:** Sociodemographic and economic characteristics of pregnant women practicing self-medication in Sodo Town, Southern Ethiopia, 2021 (*n* = 425).

Variable	Category	Frequency	Percentage (%)
Woman’s age	18–24	64	15.1
	25–29	212	49.9
	30–34	137	32.2
	35–39	12	2.8
Marital status	Married	402	94.6
	Widowed	4	0.94
	Others*	23	5.4
Women’s level of education	Primary	156	36.7
	Secondary	132	31.1
	Diploma and above	137	32.2
Husband’s education	Primary	114	26.8
	Secondary	175	41.2
	Diploma and above	136	32
Occupation	Government employee	128	30.1
	Self-employed	131	30.8
	Housewife	162	38.1
	Others^#^	4	0.9
Religion	Orthodox	131	30.8
	Muslim	80	18.8
	Protestant	201	47.3
	Others^@^	12	3.1
Wealth index	Poorest	74	17.4
	Poor	112	26.4
	Medium	54	12.7
	Rich	101	23.8
	Richest	84	19.7

*Others: single and divorced, ^#^Others: catholic, not specified, ^@^Others, not specified.

### Obstetric-related characteristics of the study participants

Among the study participants, 302 (71.1%) of the pregnant women were multigravida. Thirty (7.1%) of the pregnant women had a previous history of abortion. Four hundred and 11 (96.7%) women had ANC follow-up for this pregnancy. Of the respondents, 335 (81.5%) started their follow-up in the first trimester of their pregnancy. At the time of the interview, 161 (39.2%) of the pregnant women had only one ANC visit. One hundred and 38 pregnant women (32.5%) experienced pregnancy-related problems, of which 80 (58.0%) sought help at a health facility (Table 2).

**Table 2:** Obstetric-related characteristics of pregnant women in Sodo Town, Southern Ethiopia, 2021 (*n* = 425).

Variables	Category	Frequency	Percentage (%)
Gravidity	Primigravida	123	28.9
	Multigravida	302	71.1
Number of living children	None alive	133	31.3
	One alive	248	58.4
	≥two alive	44	10.4
Gestational age of pregnancy	First trimester	73	17.2
	Second trimester	226	53.2
	Third trimester	126	29.6
Time of first ANC visit	First trimester	335	81.5
	Second trimester	76	18.5
Number of ANC visits	One	161	39.2
	Two	153	37.2
	Three	44	10.7
	Four and above	53	12.9
Pregnancy-related problem	No	287	67.5
	Yes	138	32.5

### Prevalence of self-medication

Among 425 pregnant women, 87 (20.5%), 95% CI: [16.7, 24.2] of them practiced self-medication during the current pregnancy. Among the drugs used for self-medication, 52 (12.3%) of the pregnant women used conventional medicine and 35 (8.2%) of the pregnant women used herbal medicine.

### Self-medication with conventional medication

In this study, 52 (12.3%) of the participants used conventional medicine for self-medication. Among them, the majority (38.5%) had prior experience with drugs. Headache was the main ailment for which conventional medicine was used (*N* = 23, 44.2%), and paracetamol was the most commonly used drug (*N* = 23, 44.2%). Husbands were the most frequently cited source of information about the drug (*N* = 16, 30.8%), followed by private community pharmacies/drug stores (*N* = 33, 63.5%) (Table 3).

**Table 3:** Self-medication among pregnant women by conventional medicine in Sodo Town, Southern Ethiopia, 2021 (*n* = 425).

Variables	Category	Frequency	Percent (%)
Have you practiced SM by CM?	Yes	52	12.3
	No	373	87.7
What makes you want to practice SM by CM?	Saving time	17	32.7
	Easily available	12	23
	Sufficient knowledge about the disease and treatment	3	5.8
	Had previous experience with the drug	20	38.5
For what types of ailments have you used SM by CM?	Headache	23	44.2
	Typhoid	10	19.2
	Cough	8	15.4
	Other	11	21.2
What is the name of the medicine that you have used for SM by CM?	Paracetamol	23	44.2
	Cough syrup	8	15.4
	I do not know	12	23.1
	Others	9	17.3
Who is your source of information for using CM?	Yourself	14	26.9
	Your husband	16	30.8
	Your friend	10	19.2
	Pharmacist	11	21.2
	Others	1	1.9
Where did you get the medicine from?	Neighbor	4	7.7
	Friends	11	21.2
	Pharmacy	33	63.5
	Others	4	7.6
What did you know about the modern medicine(s) that you used?	How to take it/them	16	30.8
	No information	35	67.3
	Others	1	1.9

Others: not specified.

### Self-medication with herbal medicine

The study showed that 35 (8.2%) of the pregnant women used herbal medicine. The most frequent condition for which HM was used was the common cold (*N* = 13, 37.1%). The main reason for the use of HM (22, 67.9%) is that it was available without a prescription. “*Feto*” was a commonly used HM (*N* = 13, 37.1%). The respondents mainly obtained information about HM from family and friends (*N* = 17, 48.6%) (Table 4).

**Table 4:** Self-medication among pregnant women with herbal medicine in Sodo Town, Southern Ethiopia, 2021 (*n* = 425).

Variables	Category	Frequency	Percentage (%)
Have you practiced self-medication with herbal medicine?	Yes	35	8.20
	No	390	91.80
What motivates you to use herbal medicine for self-medication?	Herbal medicines are more effective than conventional medicines	8	22.90
	Herbal medicines have a lower cost	5	14.30
	Herbal medicines are available without a prescription	22	62.80
For what purposes and ailments have you used herbal medicines?	Headache	7	20.00
	Nausea/vomiting	4	11.40
	Common cold	5	14.30
	Diarrhea	2	5.70
	To prevent abortion	3	8.60
	Other	14	40.0
What types of herbs have you utilized?	*Feto* (*Lepidium sativum* L.)	13	37.20
	*Damakese* (*Ocimum lamiifolium*)	11	31.40
	*Kosso* (*Hagenia abyssinica*)	6	17.10
	Ginger^*^	5	14.30
Who is your source of information on herbal medicines?	Family and friends	17	48.60
	Neighbors	16	45.70
	Others	2	5.70

Others: not specified. ^*^Ginger, Nech bahirzaf.

### Factors associated with self-medication

Based on multivariable logistic regression analysis, this study found that gestational age (first and second trimesters of pregnancy) and previous history of self-medication were significantly associated with self-medication practice among pregnant women. The results indicated that pregnant women in their first and second trimesters were 3.47 and 2.14 times more likely to practice self-medication, respectively (AOR = 3.47, 95% CI: 1.63, 7.38, and AOR = 2.14, 95% CI: 1.12, 4.08), compared to those in their third trimester. Moreover, pregnant women with a history of self-medication were 6.31 times more likely (AOR = 6.31, 95% CI: 4.89, 9.91) to engage in self-medication compared to those without such a history (Table 5).

**Table 5:** Factors associated with self-medication practice among pregnant women in Sodo Town, Southern Ethiopia in 2021 (*n* = 425).

Variables	Self-medication practice		COR (95% CI)	AOR (95% CI)	P-value
	No	Yes			
Wealth index					
Poorest	29	45	10.29 (4.68,22.62)	1.91 (0.78,4.65)	0.153
Poor	103	9	0.58 (0.22,1.47)	1.78 (0.76,4.13)	0.178
Medium	45	9	1.32 (0.51,3.45)	1.50 (0.66,3.39)	0.324
Rich	88	13	0.98 (0.41,2.31)	1.56 (0.62,3.90)	0.340
Richest	73	11	1.00	1.00	
Parity					
Para zero	100	34	0.94 (0.44,1.98)	0.55 (0.01,31.06)	0.775
Para one	202	40	0.54 (0.26,1.12)	0.48 (0.02,5.51)	0.790
Para two and above	36	13	1.00	1.00	
Previous history of abortion					
Yes	21	9	1.74 (0.76,3.95)	0.46 (0.09,2.32)	0.355
No	317	78	1.00	1.00	
Gestational age in trimesters					
1st trimester	51	22	3.19 (1.53,6.65)	**3.47 (1.63,7.38)**	0.001
2nd trimester	176	50	2.10 (1.12,3.92)	**2.14 (1.12,4.08)**	0.020
3rd trimester	111	15	1.00	1.00	
Number of ANC visits					
One	119	42	1.00	1.00	
Two	118	35	0.84 (0.50,1.40)	1.45 (0.75,6.63)	0.075
Three and above	89	8	0.25 (0.09,0.84)	1.75 (0.49,5.75)	0.139
Do you have any pregnancy-related problems?					
Yes	63	75	1.00	1.00	
No	275	12	0.03 (0.19,0.71)	1.097 (0.19,6.24)	0.917
Previous history of SM practices					
Yes	60	51	6.56 (4.13, 9.15)	**6.31 (4.89, 9.91)**	< 0.001
No	278	36	1.00	1.00	

The bold values indicate statistically significant variables with a *p*-value less than 0.05.

## Discussion

This study identifies the prevalence of self-medication practices among pregnant women in Sodo Town and the factors associated with it. Although SMP is effective in treating minor ailments, improper SMP can have fatal consequences for both the mother and the fetus, in addition to serious drug reactions. This study found that 20.5% of pregnant women practice self-medication during their current pregnancy without seeking the advice of medical professionals, whether using modern or herbal medicine. This finding was in line with studies conducted in Nekemte (21.2%) and Jimma (20.1%) (26, 27). The prevalence of self-medication practice in our study was higher compared to reports from Goba (15.5%) and Bangladesh (12.2%) (24, 33). Additionally, this result was higher than studies conducted in Addis Ababa (26.6%), Gonder (44.8%), Jimma (27%), and Harar (69.4%) (2, 11, 20, 22). The possible reasons for the difference could be attributed to variations in socioeconomic factors, geographic location, access to healthcare facilities, cultural beliefs, attitudes toward healthcare providers, waiting times at healthcare facilities, affordability, survey design, sampling techniques, study settings, and sample size. Another reason could be the drug laws of the country or the electiveness of the drug regulatory agencies of the countries where the studies were conducted. Ethiopia, like the majority of Sub-Saharan African (SSA) countries, has a high workload for healthcare workers; as a result, healthcare providers may not provide adequate health education regarding the possible risks of self-medication practices on the health of pregnant women and their fetuses.

In this study, the most commonly used herbs among pregnant women were feto (*Lepidium Sativum* L), damakese (*Ocimum lamiifolium*), and kosso (*Hagenia abyssinica*). Furthermore, it was found that paracetamol was the most frequently utilized conventional medicine for self-medication. These findings indicate that pregnant women rely on both traditional herbs and modern pharmaceuticals for self-medication during pregnancy.

The odds of practicing self-medication were 3.47 and 2.14 times higher in the first and second trimesters of pregnancy, respectively than in the third trimester. This study was consistent with studies conducted in Tanzania and Ghana (10, 25, 28). This may be due to the occurrence of many symptoms and illnesses such as nausea or vomiting, headache, dizziness, and fever in the early trimesters of pregnancy, which may account for higher self-medication rates. Another possible explanation is that the initial discomfort associated with a new pregnancy and common symptoms related to pregnancy are frequent during the early stages of pregnancy, due to various physiological changes, including hormonal fluctuations.

Pregnant women with a history of self-medication were 6.31 times more likely to practice self-medication during pregnancy than those who had no prior history of self-medication. This study agreed with studies conducted in Kemisie, Wolayita Sodo, Iran, the Eastern Democratic Republic of the Congo, Nigeria, and Jimma (18, 27, 29–32). This may be due to previous drug exposure, complications during pregnancy, or a lack of knowledge about the dangers of self-medication. Another factor may be that over-the-counter (OTC) medications are seen to be safe for use during pregnancy because they are frequently used outside of pregnancy.

### Strengths and limitations of the study

#### Strengths of the study

The following are the potential areas of strength of this study. This study used a relatively large sample size. This increases the statistical power of the study and improves the generalizability of the finding to the target population. The study collected data on various obstetric-related characteristics of the study participants, including gravidity, ANC follow-ups, gestational age, number of ANC visits, and pregnancy-related problems. This comprehensive data collection allows for a thorough analysis of the factors associated with self-medication practices during pregnancy. The study examined self-medication practices with both conventional and herbal medicine. This provides a more complete understanding of the prevalence and patterns of self-medication among pregnant women, considering the use of both types of medications.

The study provided both percentage and frequency data for various variables, allowing for a clear understanding of the prevalence and distribution of self-medication practices among pregnant women. This facilitated the comparison and interpretation of the results. The study reported confidence intervals for the prevalence of self-medication practices, providing a measure of the precision of the estimates. This helped assess the reliability of the findings and the level of uncertainty associated with the prevalence estimates. The study also provided detailed information on the reasons for self-medication, the types of ailments for which self-medication is practiced, the specific medications used, and sources of information about the medicines. This level of detail allowed for a deeper understanding of the motivations and patterns of self-medication among pregnant women.

The study focused on Sodo Town in Southern Ethiopia, providing insights into the self-medication practices of pregnant women in this specific region. This localized approach allowed for a more targeted understanding of the factors influencing self-medication practices in a specific context. The study was conducted in 2021, which ensured that the data collected was relatively up-to-date, reflecting current practices and trends in self-medication among pregnant women in the study area. The study findings could serve as a basis for further research and interventions to improve the safe and appropriate use of medications during pregnancy. The identified strengths of the study could guide future studies to explore related topics or expand the scope of the research.

#### Limitations of the study

The study has several limitations that should be considered when interpreting the findings. These limitations include using a cross-sectional design, which only provided a snapshot of self-medication practices at a specific time and did not allow for the establishment of causal relationships or the assessment of changes over time. The reliance on self-reported data introduced the potential for recall and social desirability bias. The limited generalizability of the study due to its specific geographic location and lack of a control group also limited the ability to generalize the findings to other populations. The study did not account for potential confounding factors that may have influenced self-medication practices, such as income level or access to healthcare services. Additionally, the study lacked detailed information on specific medications and their dosages in addition to the duration and frequency of self-medication practices. The absence of qualitative data and information on adverse outcomes further limited the understanding of the motivations, beliefs, and experiences of pregnant women regarding self-medication practices and their potential impact on maternal and fetal health outcomes.

## Conclusion and recommendations

Self-medication is a common practice among pregnant women in Sodo Town, Southern Ethiopia. This study found that factors associated with self-medication included a previous history of self-medication and gestational age. These findings underscore the need for targeted educational programs and policy interventions to mitigate the risks associated with self-medication during pregnancy, particularly in the early stages of gestation. To address this issue, we recommend implementing health education programs that focus on the risks of self-medication during pregnancy. These programs should involve local health authorities, healthcare providers, and community leaders to ensure comprehensive outreach. Continuous monitoring and evaluation of these programs is essential to assess effectiveness and make necessary adjustments. By addressing these factors, we can reduce the prevalence of self-medication and improve maternal health outcomes.

## Data Availability

The data set generated and/or analyzed during the current study is not publicly available due to preserving participant anonymity but is available from the corresponding author upon reasonable request.
